# Paired design study by real-time PCR: miR-378* and miR-145 are potent early diagnostic biomarkers of human colorectal cancer

**DOI:** 10.1186/s12885-015-1123-2

**Published:** 2015-03-21

**Authors:** Juan Peng, Zhengyong Xie, Liyang Cheng, Yuxin Zhang, Junyong Chen, Hongping Yu, Zehang Li, Huixing Kang

**Affiliations:** 1 Guilin Medical College, Huang Cheng North 2 Road 109, Qixing District, Guilin, Guangxi China; 2 General Hospital of Guangzhou Military Command of PLA, Liuhua Road 111, Guangzhou, Guangdong China

**Keywords:** MiRNA, Colorectal cancer, Early diagnosis

## Abstract

**Background:**

Although microRNAs offer great potential as cancer biomarkers, effective clinical dignostics and tumor maker have not been verified to diagnose with colorectal cancer (CRC). The purpose of our study is to systematically assess the expression of miRNAs in matched cancer and normal tissue samples to identify promising diagnostic microRNA (miRNA) biomarkers for CRC.

**Methods:**

In our study, we examined by Real-Time PCR the expression levels of 96 mature miRNA in 32 CRC patients with differently expressed tumors versus normal colon tissues. Using enter and stepwise variable selection methods separately, conditional logistic regression was conducted to identify miRNAs associated with CRC. The classification performance of these indicators was assessed under the Fisher discriminant analysis. Receiver operating characteristic curve analyses were applied to obtain diagnostic utility of the differentially expressed miRNAs.

**Results:**

In this study, we confirmed 11 overexpressed miRNAs with no less than twofold difference, and 85 downexpressed miRNAs with up to 0.5-fold difference in CRC from 96 aberrantly expressed miRNAs being identified by real-time PCR. Conditional logistic regression results confirmed that miRNA-378 and miRNA-145 expression profile was statistically significant. The error diagnosis rate of these two miRNAs are 0.194 and 0.113, separeately, showing by discriminant analysis.

**Conclusions:**

MiRNA-145 and miRNA-378* are potential biomarkers for early detection of CRC, which may help in diagnosing CRC in early period.

## Background

Colorectal cancer (CRC) yields the second highest mortality rate in China, showing a high incidence trend. Early diagnosis and treatment of CRC is an effective way to improve patients’ survival. However, most cases are diagnosed with CRC at late stages as current clinical diagnostics and tumor markers are inconvenient and population screening rates are low. Therefore, there is an imperative need to search for specific, sensitive biomarkers for the early diagnosis of CRC.

MiRNA is small, non-coding sighle-strand RNA, which contain of about 19 nucleotides to 25 nucleotides arising from one arm of longer endogenous hairpin transcripts. Growing studies suggests that microRNA (miRNA) plays an important role in colon disease process, including cell differention, development, proliferation and translation. Futhermore, misregulation of miRNA expression might contribute to human diseases. Evidence has shown that miRNA can work with the target mRNA 3′ non-transcribed with incomplete or complete pairing and negatively regulate gene expression in post-transcriptional level by degrading the target mRNA or inhibit translation [1].

There is increasing evidence that some miRNAs may be used as diagnostic biomarkers for CRC by identifying differences in miRNA expression of colorectal tumors and ajacent non-neoplastic tissues from patients. For example, E Bandrés et al. reported that miR-31, miR-96, miR-133b, miR-135b, miR-145, and miR-183 are the most significantly deregulated miRNAs and the expression level of miR-31 was correlated with the stage of CRC tumor [2]. Calin et al. reported that one of the most upregulated miRNAs is miR-106a, which is consistently reported in six studies, and the five most downregulated miRNAs are miR-30a-3p, miR-139, miR-145, miR-125a, and miR-133a, which are consistently reported and differentially expressed in four studies; these miRNAs may actually be of clinical use as diagnostic/prognostic biomarkers or therapeutic targets [3]. However, most of the screening studies have small sample with no prediction accuracy of candidate diagnostic biomarkers.

To determine much sensitive indicators, the present study examined the expression of 96 mature miRNAs in a panel of 31 matched pairs of tumoral and non-tumoral tissues by real-time PCR. Conditional logistic regression was used to screen the factors contributed to the occurrence of tumor, among which miR-145 and miR-378* were found being statistically significant. Futher analysis reveals miR-378* and miR-145 exhibits potential as a good diagnostic and prognostic marker.

## Methods

To investigate whether miRNAs are differentially expressed in CRC versus normal colon tissues, surgical specimens of cancer tissue and adjacent normal mucosa were obtained from 32 patients with colorectal cancer who underwent surgery at The General Hospital of PLA Guangzhou Military Area between 2012 and 2013. Fresh CRC and adjacent noncancerous colorectal tissues, which were used as specimens, were obtained by experienced surgeons and examined by experienced pathologists. Surgery was performed to remove the primary tumor immediately after carcinoma was diagnosed. Incision was performed from the edge of the site by at least 2 cm. Tumor stage was classified according to the International Union against Cancer (UICC, 6th ed., 2002). Informed written consent was obtained from each patient, and research protocols were approved by the Medical Ethics Committee of the General Hospital of PLA Guangzhou Military Area.

RNA extraction was performed to obtain cryopreserved tissues and adjacent group-woven sliced tissues with liquid nitrogen, grinding into powerder using Trizol total RNA isolation reagent as per the manufacturer’s protocal [4]. Concentration and purity of isolated RNA were assessed by measuring the optical density at 260 (OD260) and 280 nm (OD280).

Isolated RNA was of high purity and integrity. A260/A280 ratios for the human genomic DNA extracted from spit were consistently within generally acceptable values of 1.7 to 2.0 [5]. Reverse transcription-related operations were performed following the kit instructions.

Quantification was performed by real-time PCR using SYBR Premix Ex Taq TM (TaKaRa) for the most upregulated or most downregulated miRNAs. The primers for U6 were obtained from TaKaRa. PCR was performed in a real-time PCR system (Bio-Rad) with the following reaction conditions: Real-time PCR kit was operated in strict accordance with the manufacturer’s instructions. The cycling program consists of 1× at 95°C for 10 min and 40× (15 s at 95°C) at 60°C, plus extension of 60 s, for a total of 40 cycles [6].

Default threshold settings were used as threshold cycle (Ct) data. The Ct is the fractional cycle number at which the fluorescence passes the fixed threshold [7]. After calculating the Ct value, expression values were normalized to those for U6, which were calculated as ΔCt = Ct − Ct_U6_ (expression of the relative expression profile). Therefore, ΔΔCt = ΔCt (tumor group) − ΔCt (control group) [8]. Relative quantification of miRNA expression was calculated with 2^-ΔΔCt^ method, which represents relative fold changes of miRNA expression.

### Statistical analysis

The patients’ demographics for continuous variables were reported as mean ± SD, while percentage, and frequency calculated for categorical variables. Paired Student’s *t*-test was used to determine the level of significance (P < 0.05). Conditional logistic regression model was used to calculate odds ratio with 95% confidence interval to estimate association and control the potential confounding variables, confirming diagnostic use of miRNAs that contributes to CRC probability. Discriminant analysis for classification of tissues types [9] was performed to determine the discriminative ability of the screened miRNAs. Statistical analysis was performed using IBM SPSS version 16.0 software. P values of less than 0.05 were considered statistically significant.

## Results

### Clinicopathological characteristics of CRC patients

The clinicopathological factors of the 32 (16 women) participants with CRC recruited to the study are showed in Table 1. The mean age of the respondents was 31.3 years (±5.6 SD). No participants showed evidence of disease complications. A total of 23 participants (71.9%) were within the age range of 60 years to 83 years, while9 patients (28.1%) were in the age range of 20 years to 60 years. In addition, 16 participants (50%) had stage II disease, while the other 16 (50%) were diagnosed with stages III and IV disease. Although Beştaş R et al. suggested that vascular endothelial growth factor (VEGF) expression is significantly correlated with advanced stage [10], we did not found any correlation in this study. Among nine patients reported VEGF, 4 patients (44.44%) were negative in VEGF and 5 out of them were positive. Loss of CK20 expression is associated with poorly differentiated carcinoma [11]. Ten (31.3%) out of 31 patients were positive in CK20.

**Table 1:** **Clinicopathological characteristics of CRC patients**

Variable	Frequency	Percentile (%)
Sex		
Male	16	50.0
Female	16	50.0
Age at diagnosis (years)		
<60	9	28.1
≥60	23	71.9
Stage		
II	16	50.0
III	13	40.6
IV	3	9.4
VEGF		
Negative (9)	4	44.44
CK20		
+	2	20.0
++	4	40.0
+++	4	40.0

### Fold change of 96 selected miRNAs further validated by TaqMan RT-qPCR

To identify miRNAs that are differentially expressed in tissues, we analyzed expression profiles of 1448 miRNAs, founding 497 miRNAs expression profiles showed statistical significance, in which 155 miRNAs have statistical significance in paired t-test and satisfied the strict condition of P ≤ 0.001.

**Figure 1:**
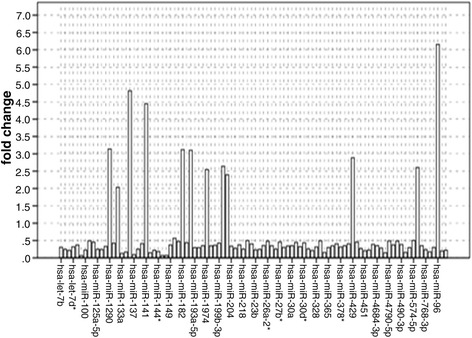
Fold change of 96 selected miRNAs further validated by TaqMan RT-qPCR. Twelve miRNAs (hsa-miR-130b, hsa-miR-203, hsa-miR-1974, hsa-miR-592, hsa-miR-200a, hsa-miR-429, hsa-miR-183, hsa-miR-182, hsa-miR-1290, hsa-miR-141, hsa-miR-135b, and hsa-miR-96) were overexpressed, whereas 84 miRNAs (hsa-miR-1, hsa-miR-145, hsa-miR-145*, and so on) were downexpressed in tumor tissues compared with those in normal tissues.

To further validate the expression levels of these miRNAs, 31 patients were qualified and selected for performing qPCR validation phase. In the condition of fold chang >2.0 and P < 0.05, we gained a set of 96 miRNAs that were differentially expressed between the colorectal tissues and normal tissues, which were consistent with the results of TaqMan Human MicroRNA Array. Twelve miRNAs were upexpressed, while 84 miRNAs (has-miR-137, hsa-miR-133a, hsa-miR-143, hsa-miR-363, hsa-miR-4770, hsa-miR-490-5p, hsa-miR-133b, and so on) were deexpressed in tumors compared with those in normal tissues (adjusted P = 0.05) (Figure 1).

### Selected miRNA expression levels in CRC tissues and neighboring noncancerous tissues

After a series of selection processes independently with enter method and conditional forward method in conditional logistic regression, we found nine statistically significant miRNAs in enter method, namely, miR574-3p, miR422a, miR490-3p, miR-374b, miR-133a, let7g, miR-378*, miR-9* and miR-378i.

**Figure 2:**
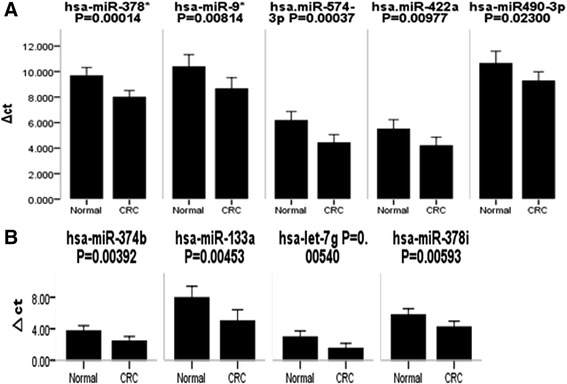
Selected miRNA expression levels in CRC tissues and neighboring noncancerous tissues. Selected miRNAs showed statistical significance in conditional logistic regression analysis. **(A)** has-miR874-3p, miR422a, and miR490-3p expression levels in CRC tissues and neighboring noncancerous colorectal tissues. **(B)** has-miR374b, has-miR133a, has-let-7 g, has-miR378, has-miR9*, and has-miR378i expression levels in CRC tissues and neighboring noncancerous colorectal tissues.

On the other hand, we found seven statistically significant miRNAs, namely, miR-145, miR-363, miR-378*, miR-137, miR-100, miR-125a-5p, miR-143 in conditional forward method. This result was consistent with Pagliu A. et al.’s study who found that analysis of the combined action of miR-143 and miR-145 on oncogenic pathways in colorectal cancer cells reveals a coordinate program of gene repression [12]. Besides, the expression levels of these miRNAs between CRC tissues and the neighboring noncancerous colorectal tissues were compared using qRT-PCR analysis. Preliminary results showed that the level of miR874-3p, miR-422a, miR-490-3p, miR-374b, miR-133a, let-7 g, miR-378, miR-9*, and miR-378i were all deregulated in the CRC tissues compared with the neighboring noncancerous colorectal tissues (all P < 0.05) (Figure 2).

### Logistic regression and fold change of miR-145 in different clinical stages

**Figure 3:**
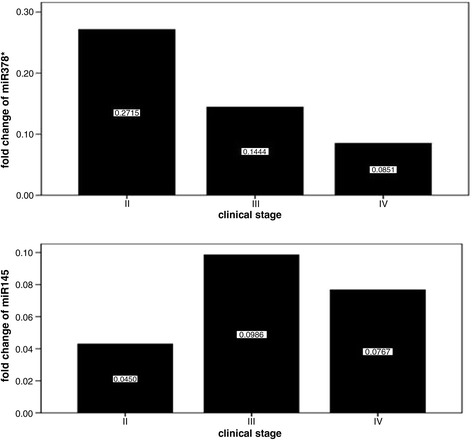
Fold change of miR-378* and miR-145 in different clinical stages. Expression levels of miR-378* and miR-145 varied in different clinical stages.

To further screen the most contribution factors to CRC among these miRNAs, the variables above were used to analyze in the conditional logistic regression. The results indicated that miR-378* is the kept variable with statistical significance to distinguish CRC from normal tissues (P < 0.05; odds ratio = 4.6; 95% CI of odds ratio = 1.25 to 16.84). Similarly,miR145 is also the kept variable with statistical significance to distinguish CRC from normal tissues (P < 0.05; odds ratio = 4.21; 95% CI of odds ratio = 1.17 to 15.13) (Table 2). These results were consistent with those from the study of Zhang et al., who indicated that miRNA378 is a reliable, hemolysis-independent biomarker for CRC [13]. We compared the expression of miR-378* in different clinical stages and found that miR-378* is downregulated in all stages. A previous study also proved that the expression of miR-378a-3p and miR-378a-5p was significantly associated with TNM stage [14]. These results indicate that clinical stage may influence the expression of miRNAs (Figure 3).

**Table 2:** **Conditional logistic regression for the relationship between miRNAs and CRC**

Variable	Coefficient	Standard error	Wald	P value	Odds ratio	95% CI
hsa-miR-378*	1.525	0.663	5.297	0.021	4.596	1.25 to 16.84
hsa-miR-145	1.438	0.653	4.853	0.028	4.210	1.17 to 15.13

### Discriminant analysis for miR-378*

To verify the diagnostic value of miR-378*, Fisher discriminant analysis was analyzed to predict the category and determine the diagnostic ability of miR-378*. After ln-gamma transformation of the sample data, the variables followed normal distributions and equal variance matrix for categories I and II.

Evidently, as shown in Table 3, true-positive rate is 80.65% and false-positive rate is 19.35%. The whole discriminant analysis results are listed in Table 3.

**Table 3:** **Discriminant analysis results**

Original group	Prediction		Total	Error rate (%)
	Tumor	Normal		
Tumor	25	6	31	19.35
Normal	6	25	31	19.35
Total	31	31	62	19.35

### Receiver operator characteristic curve for miR-378* and miR-145

**Figure 4:**
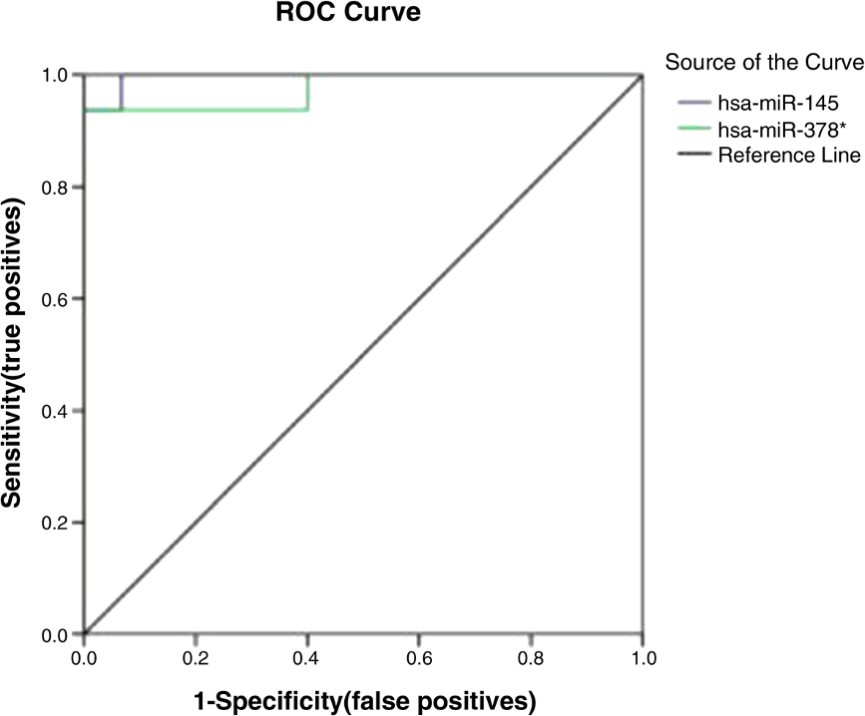
Receiver operator characteristic curve for miR-378* and miR-145 for the predictor of CRC.miRNA-378* levels had areas under the receiver operator characteristic curve of 97.5% and 99.6% separately.

Area under the curve for the receiver operator characteristic function for miR-378* and miR-145 were 0.975 (95% confidence interval 0.923–1.00, p < 0.0005) and 0.996 (95% confidence interval 0.982–1.00, p < 0.0005) separately. At an optimal cut score of ≥3.80 and < −1.975(fold change 0.025) separately, sensitivity was 100% both and specificity was 60% and 98% separately. A score of −1.167 (fold change 0.833) or greater for miR-145 had 93.3% specificity but had sensitivity of only 93.8% (See Figure 4, Table 4).

**Table 4:** Coordinates of the ROC Curve for miR378* and miR145

miR378*			miR145		
Cut-off value	Sensitivity (%)	Specificity (%)	Cut-off value	Sensitivity (%)	Specificity (%)
37.90	100	0.0	−11.084	100	0.0
12.52	100	7.7	−8.427	100	0.1
7.02	100	13.3	−6.117	100	0.1
5.89	100	20.0	−5.166	100	0.2
5.70	100	16.7	−4.489	100	0.3
5.28	100	33.3	−3.919	100	0.3
4.93	100	40.0	−3.717	100	0.4
4.31	100	46.7	−3.390	100	0.5
3.82	100	53.3	−3.063	100	0.5
3.80	100	60.0	−2.864	100	0.6
3.43	93.8	60.0	−2.495	100	0.7
2.39	93.8	66.7	−2.294	100	0.7
1.82	93.8	73.3	−1.975	100	0.8
			−1.167	93.8	0.9
			−0.082	93.8	1.0

## Discussion

Interestingly, our results in CRC samples agree with those obtained by Faltejskova P et al., who found that the miRNAs functioning as tumor suppressors in CRC are miR-378, miR-375, miR-422a, and miR-215 from 667 miRNAs in a paire design study [15]. Furthermore, Cui SY et al. found that microRNA-145 as a potent tumour suppressor that regulates multiple cellular pathways [16]. Zhang GJ et al. also indicated that miR-378 is an independent prognostic factor that inhibits cell growth and invasion in CRC. Most important is HouY et al. suggest that microRNA-145 as ideal biomarker for the diagnosis of various carcinomas [17].

Notably, our current study showed that discriminant analysis based on expression data revealed that miR-378* can distinguish CRC from normal tissues. This result is important for the potential role of new molecular gene miR-378* in future diagnostic processes in the absence of effective early diagnostic biomarkers.

The different biological effects of any particular miRNA in different cells could be dependent of the cell-specific collection in target genes [18]. miR-378 inhibits progression of human gastric cancer MGC-803 cells by targeting MAPK1 in vitro [19]. miR-378 also promotes BMP2-induced osteogenic differentiation of mesenchymal progenitor cells [20]. Furthermore, miRNA-profiling study proved that miR-378 was significantly upregulated in cachexia related to enhanced adipocyte lipolysis in human cancer [21]. Notably, Panza A, et al., found that miR-145 is a novel target of PPARγ, acting as a tumor suppressor in CRC cell lines and being a key regulator of intestinal cell differentiation by directly targeting SOX9, a marker of undifferentiated progenitors in the colonic crypts [22].

Therefore, high-field miR-378* and miR-145 are powerful means for investigating early CRC signs and are promising sensitive tools to support medical diagnosis. Future validation of these methods is preparing planned on a prospective study. Thus, previous studies reveals that change of miR-378 expression has been previously reported in nasopharyngeal cancer. Yu BL et al. reported that repressing TOB2 expression would cause miR-378 to function as an oncomiR in nasopharyngeal carcinoma [23]; miR-378/ATP also binds Cassette Transporter G1-Signaling pathway [24].

## Conclusions

In summary, our real-time PCR results identified alterations of miRNA expression in CRC with two down-regulated miRNAs (miR-378* and miR-145), which may be novel candidate biomarkers for CRC. Although, both miR-145 and miR-378* contribute to the probability of CRC, comparison between the roles of miR-145 and miR-378* in the CRC progression will give more information to the cell transcription mechanisms.

Further mechanistic studies focusing on miR-378* and miR-145 are required to investigate its underlying roles in the tumorigenesis of CRC [24].

## Abbreviations

CRC: Colorectal cancer
Real-time RT-PCR: Real-time reverse transcription polymerase chain reaction
